# Recent Dental Patents

**Published:** 1859-08

**Authors:** 


### RECENT DENTAL PATENTS.

BASES FOR ARTIFICIAL TEETH—Geo. Dieffenbach, of New York, N. Y.: I claim the composition of matter, consisting of sulphate of alumina and other ingredients, substantially as described for the purpose as set forth.

PROCESS FOR COLORING ARTIFICIAL TEETH—Geo. Dieffenbach, of N. Y.: I claim developing the color of a cured or hardened composition, by the agency of solar light, when the coloring matter is incorpoated into the said composition, while in its plastic or uncured state, substantially as described.

